# Impact of Kefiran Exopolysaccharide Extraction on Its Applicability for Tissue Engineering and Regenerative Medicine

**DOI:** 10.3390/pharmaceutics14081713

**Published:** 2022-08-17

**Authors:** Susana Correia, Cristiana Gonçalves, Joaquim M. Oliveira, Hajer Radhouani, Rui L. Reis

**Affiliations:** 1 3B’s Research Group, I3Bs—Research Institute on Biomaterials, Biodegradables and Biomimetics, University of Minho, Headquarters of the European Institute of Excellence on Tissue Engineering and Regenerative Medicine, AvePark, Parque de Ciência e Tecnologia, Zona Industrial da Gandra, Barco, 4805-017 Guimarães, Portugal; 2 ICVS/3B’s–PT Government Associate Laboratory, Braga, 4805-017 Guimarães, Portugal

**Keywords:** characterization, extraction, kefiran, regenerative medicine, scaffolds, tissue engineering

## Abstract

Kefiran is an exopolysaccharide produced by the microflora of kefir grains used to produce the fermented milk beverage kefir. The health-promoting and physicochemical properties of kefiran led to its exploration for a range of applications, mainly in the food industry and biomedical fields. Aiming to explore its potential for tissue engineering and regenerative medicine (TERM) applications, the kefiran biopolymer obtained through three different extraction methodologies was fully characterized and compared. High-quality kefiran polysaccharides were recovered with suitable yield through different extraction protocols. The methods consisted of heating the kefir grains prior to recovering kefiran by centrifugation and differed mainly in the precipitation steps included before lyophilization. Then, kefiran scaffolds were successfully produced from each extract by cryogelation and freeze-drying. In all extracts, it was possible to identify the molecular structure of the kefiran polysaccharide through ^1^H-NMR and FTIR spectra. The kefiran from extraction 1 showed the highest molecular weight (~3000 kDa) and the best rheological properties, showing a pseudoplastic behavior; its scaffold presented the highest value of porosity (93.2% ± 2), and wall thickness (85.8 µm ± 16.3). All extracts showed thermal stability, good injectability and desirable viscoelastic properties; the developed scaffolds demonstrated mechanical stability, elastic behavior, and pore size comprised between 98–94 µm. Additionally, all kefiran products proved to be non-cytotoxic over L929 cells. The interesting structural, physicochemical, and biological properties showed by the kefiran extracts and cryogels revealed their biomedical potential and suitability for TERM applications.

## 1. Introduction

Among the natural polymers, polysaccharides have emerged as promising materials for tissue engineering (TE) applications. Over the last decades, the development of advanced functional materials with outstanding properties has been the subject of intense research due to the increasing demand of new materials for biomedical applications [1]. For tissue engineering applications, these materials should act as analogues of the native extracellular matrix, by maintaining the cells’ desired phenotype and the proper function. Furthermore, the biomaterials should be biocompatible and have corresponding mechanical properties to those of the new regenerated tissues [2].

During the last decades, polysaccharide-based materials have been used in different forms, such as injectable hydrogels or fibrous and porous scaffolds to engineer suitable tissues. In fact, polysaccharides have been applied for TE purposes as coatings and delivery systems, wound healing, and scaffolds, among others. These polymers are considered attractive materials because of their cytocompatibility, biodegradability, high bioavailability, and their natural abundance [3]. Their chemical structures are characterized by the presence of hydrophilic groups, such as hydroxyl, amino, carboxylic acids, and sulfate groups that will enhance the bio-adhesion properties through non-covalent bonds toward biological tissues and growth factors [4]. Thus, it is important to point out that these polysaccharides also exhibit overwhelming structural, physical, and biological properties.

The most common polysaccharides applied in tissue engineering are alginate, agar, cellulose, chitosan, dextran, gellan, glycosaminoglycans, hyaluronic acid, pectins, pullulan, starch, and xanthan, among others. These biopolymers have been explored to repair and regenerate several tissues such as articular and tracheal cartilage, blood vessels, bone, intervertebral discs, menisci, skeletal muscle, skin, among others. They were also used to encapsulate and deliver ovarian follicles and pancreatic islets [5].

Kefir is an ancient beverage produced by fermenting milk with kefir grains, a complex and highly variable symbiotic consortium of lactic acid bacteria, acetic acid bacteria and yeasts, together with casein and complex sugars in a polysaccharide matrix [6,7]. Kefiran is the main exopolysaccharide produced by the microflora of kefir grains, being mostly produced by *Lactobacillus kefiranofaciens* [8,9]. It is a water-soluble branched glucogalactan heteropolysaccharide with nearly equal amounts of glucose and galactose with numerous beneficial properties such as antimicrobial, antioxidant, antitumor, immunomodulatory, and anti-inflammatory properties, also having antidiabetic, anti-hypercholesterolemic, and antihypertensive effects, among others [10]. These characteristics, together with its physicochemical properties and recognized safe status, have led to the exploration of kefiran for a range of applications, mainly in the food industry and biomedical fields [10,11,12]. Kefiran has been receiving increasing interest due to its unique features regarding rheological behavior, biodegradability, biocompatibility, safety, emulsifier effect, stabilizing effect, resistance against hydrolysis, barrier, and mechanical properties, and water vapor permeability [8,11,13]. Moreover, as an inexpensive biopolymer, kefiran also represents a valuable green material that can have an important contribution to achieve different Sustainable Development Goals (SDG), not only by significantly diminishing the cost of biomedical research but also by reducing the carbon footprint of the biomedical industry as it can represent an alternative to reduce the use of non-biodegradable and non-renewable materials [10,14]. In addition, the commercial value of kefiran is greatly increasing with the worldwide ever-growing and insisting demand among health and environmentally conscious consumers [10].

The applicability of the kefiran polysaccharide for tissue engineering and regenerative medicine has been previously explored by Radhouani et al. [9,15,16,17], and others [11,18,19,20,21,22,23] as viscosupplementation, films and scaffolds, coatings, drug delivery systems, among other TERM applications. However, distinct kefiran extraction methods are usually performed which are reported to differently affect the obtained results [24].

The extraction is considered a critical first step, where the required biomolecule is separated from the raw materials, while keeping its bioactivity intact. In fact, the extraction of natural bioactive polysaccharides, such as kefiran, with high purity, and with maximum extraction yield, meanwhile keeping in view that the native structure remains intact, are of great future concern, and remains a field for further exploration [25,26]. Furthermore, it has been demonstrated that the physicochemical and biological properties of natural polysaccharides vary according to the raw material and extraction method used, and the potential of bioactive polysaccharide application is highly influenced by their purity, configuration, chemical structure, and molecular weight [27].

Here, our research work aimed to optimize the kefiran isolation process, to fully characterize and compare the biopolymer obtained through three different extraction methodologies, and to explore its potential for TERM applications. Thus, kefiran exopolysaccharide extracts were obtained through three different procedures and extensively characterized by ^1^H nuclear magnetic resonance spectroscopy (NMR), Fourier transform infrared spectroscopy (FTIR), gel permeation chromatography–size exclusion chromatography (GPC-SEC), differential scanning calorimetry (DSC), and by injectability and rheological assays. Further, 3D scaffolds were produced by cryogelation and characterized by rheology, scanning electron microscopy (SEM), and micro-computed tomography (micro-CT). In addition, the in vitro cytotoxicity screening of the developed cryogels was evaluated to assess its potential as a scaffold for application in different tissue engineering and regenerative medicine strategies.

## 2. Materials and Methods

### 2.1. Kefiran Extraction

A triplicate amount of 20 g of kefir grains, previously kept in continuous skimmed milk culture at room temperature with shaking, were harvested and washed with ultrapure water to initiate three different kefiran extraction methods.

#### 2.1.1. Method 1 (M1)

M1 was performed as previously described by Radhouani, Goncalves, Maia, Oliveira, and Reis [9]; the kefir grains were heated in 200 mL of ultrapure water at 80 °C for 30 min. After centrifugation at 18,300× *g* for 20 min at 20 °C, the exopolysaccharide (EPS) in the supernatant was precipitated overnight in two volumes of cold absolute ethanol at −20 °C. After new centrifugation at 18,300× *g* for 20 min at 4 °C, the pellets were dissolved in ultrapure water at 60 °C and an additional round of ethanol precipitation was performed as described. The pellets were then dissolved in ultrapure water at 60 °C and concentrated for a crude polysaccharide by freeze-drying.

#### 2.1.2. Method 2 (M2)

M2 was performed as M1 but with the inclusion of a step of trichloroacetic acid (TCA) precipitation of contaminating material (proteins, polypeptides) before ethanol precipitation. The kefir grains were heated at 80 °C for 30 min in 200 mL of ultrapure water. After centrifugation at 18,300× *g* for 20 min at 20 °C, 20 mL of 80% (*w/v*) TCA at 4 °C was added to the supernatants and the mixture was left overnight at 4 °C. After new centrifugation at 18,300× *g* for 20 min at 4 °C, the supernatants were recovered, and two rounds of ethanol precipitation were performed as described for M1. The pellets were then dissolved in ultrapure water at 60 °C and concentrated for a crude polysaccharide by freeze-drying.

#### 2.1.3. Method 3 (M3)

M3 was performed as previously described by Pop, Salanţă, Rotar, Semeniuc, Socaciu, and Sindic [24], with slight modifications. The kefir grains were initially placed at −20 °C for a minimum of 24 h. Then, the frozen kefir grains were added to 200 mL of hot ultrapure water, heated at 80 °C for 30 min, left to cool, and centrifuged for 10 min at 10,000× *g* and 4 °C. For this method, an EPS purification procedure was performed twice where the supernatant was kept overnight at −20 °C, slowly thawed at room temperature, and centrifuged at 5000× *g* for 10 min at 4 °C. The pellets were then dissolved in ultrapure water at 60 °C and concentrated for a crude polysaccharide by freeze-drying.

### 2.2. Production of Kefiran Scaffolds

Kefiran cryogels were produced as previously described by Radhouani, Bicho, Goncalves, Maia, Reis, and Oliveira [16]. Briefly, 2% (*w/v*) kefiran solutions were prepared in ultrapure water for each extract. A volume of 200 µL was then distributed into 96-well plate molds that were immediately placed at −20 °C for 24 h and at 4 °C for an additional 24 h. Finally, the kefiran cryogels in the microplates were freeze-dried at −77.7 °C and 0.035 mbar (Telstar LyoAlfa 10/15) for 7 days.

### 2.3. ^1^H Nuclear Magnetic Resonance Spectroscopy (NMR)

To elucidate the kefiran molecular structure of each kefiran extract, 700 µL of 1% (*w/v*) solutions prepared in deuterium oxide (Sigma Aldrich, St. Louis, MI, USA) and heated for 15 min at 40 °C were transferred to an NMR sample tube. ^1^H-NMR spectra were recorded on a Varian Unity Plus (Varian, Palo Alto, CA, USA) spectrometer at 60 °C and a resonance frequency of 400 MHz. Chemical shifts were reported in ppm (δ) and MestReNova Software 9.0 (Mestrelab Research, La Coruña, Spain) was used for spectral processing.

### 2.4. Fourier Transform Infrared Spectroscopy (FTIR)

The transmission spectra of each kefiran extract were acquired by placing the samples directly on an IRPrestige-21 spectrometer (Shimadzu, Kyoto, Japan) and analyzed through the IRsolution software using 32 scans, a resolution of 4 cm^−1^, and a wavelength range between 4000 and 600 cm^−1^.

### 2.5. Gel Permeation Chromatography–Size Exclusion Chromatography (GPC-SEC)

The molecular weight (Mw) of the different kefiran extracts was determined by performing GPC-SEC of 1 mg/mL solutions prepared in phosphate-buffered saline (0.01 M phosphate buffer, 0.0027 M potassium chloride, and 0.137 M sodium chloride, pH 7.4, at 25 °C, Sigma-Aldrich, St. Louis, MI, USA) and 0.05% *w/v* NaN_3_ at rate of 1 mL/min. GPC measurements were performed with a Malvern Viscotek TDA 305 with refractometer (RI-Detector 8110, Bischoff), right- and low-angle light scattering (LS), and viscometer detectors on a set of four columns: pre-column Suprema, 5 µm, 8 × 50, Suprema 30 Å, 5 µm, 8 × 300, and 2× Suprema 1000 Å, 5 µm, 8 × 300. The system was kept at 30 °C. The absolute molecular weight was determined by a calibration of the Refractive Index (RI) and Light Scattering (LS) detectors performed using the software Omnisec 5.12 (ViskoteK) with a pullulan number-average molecular weight (Mn) of 48.8 kDa and a polydispersity index (PDI) of 1.07. The refractive index increment (dn/dc) specific of the polysaccharide was set to 0.15.

### 2.6. Differential Scanning Calorimetry (DSC)

DSC experiments were performed in a TA-Q100 equipment under a dry nitrogen atmosphere at a flow rate of 50 mL/min. A total of 7 mg of each kefiran extract was packed into aluminium pans and an empty pan was used as reference. Each sample was heated from 20 °C to 200 °C at a constant rate of 20 °C/min, left isothermal for 2 min, cooled at the same rate to the initial temperature, and left isothermal again for 2 min before initiating a second heating run. The TA Universal Analysis 2000 software was used to determine the onset of melting temperatures, peak temperatures, and transition enthalpies (ΔH).

### 2.7. Injectability (Extrusion) Assay

The injectability of the different kefiran extracts was assessed in 1% (*w/v*) solutions prepared in ultrapure water. An injection measurement device (KD Scientific, Holliston, MA, USA) consisting of a syringe pump with a 1 mL plastic syringe and a needle gauge of 21 was used working in extrusion mode with a 1 mL/min rate. The injectability of water was measured as a reference.

### 2.8. Rheology

Rheological analyses were performed in a Kinexus Pro+ Rheometer (Malvern Instruments, Malvern, UK), using the acquisition software rSpace. The measuring system was equipped with a stainless steel (316 grade) cone-plate system with an upper measurement geometry cone, 40 mm of diameter and 4°, and a lower plate pedestal. To obtain shear viscosity and shear stress as a function of the shear rate, rotational experiments from 0.1 s^−1^ to 100 s^−1^, at 37 °C, were performed for each kefiran extract in 1% (*w/v*) solutions prepared in ultrapure water. Oscillatory experiments were performed on the kefiran scaffolds to study their viscoelasticity though frequency sweep curves obtained at 37 °C from 0.01 Hz to 1 Hz with a shear strain of 1.3%. All plots were obtained by the average of at least three experiments. Through oscillatory experiments, the average mesh size (*ξ*, nm) and the crosslinking density (*n_e_*, mol/m^3^) of the scaffolds were determined [28]. The mesh size is defined as the distance between the crosslinking points that can be established by the rubber elastic theory (RET), Equation (1):

(1) $$\xi ={\left(\frac{G^{\prime }NA}{RT}\right)}^{-1/3}$$

where *G*’ is the storage modulus, *N_A_* is the Avogadro constant (6.022 × 10^23^), *R* represents the molar gas constant (8.314 J/K mol), and *T* is the temperature in Kelvin (25 °C = 298.15 K) [29]. The values obtained with these units are in meters and then converted to nanometers. The *n_e_* (mol/m^3^) is nominated by the number of elastically active connection points in the network per unit of volume, calculated by RET, Equation (2):

(2) $$n_{e}=\frac{G_{e}}{RT}$$

where *G_e_* is the plateau value of the storage modulus measured by the frequency sweep test [30].

### 2.9. Scanning Electron Microscopy (SEM)

To analyze the kefiran scaffolds by SEM, intact and longitudinally and transversally cut scaffolds were attached to aluminum stubs using carbon tape and coated with platinum in a sputter coater (Model EM ACE600, Leica, Wentzler, Germany). Morphology images were obtained on a JSM-6010LV scanning electron microscope (JEOL, Tokyo, Japan), featuring integrated energy dispersive spectroscopy (INCAx-Act, PentaFET Precision, Oxford Instruments, Santa Barbara, CA, USA).

### 2.10. Micro-Computed Tomography (Micro-CT)

The microstructure of the kefiran scaffolds was evaluated using a high-resolution X-ray microtomography system Skyscan 1272 scanner (Bruker Micro-CT, Kontich, Belgium) with a defined pixel size of 5 μm, a 2452 × 1640 resolution, and a rotation step of 0.45° over a rotation range of 360°. Acquisitions were performed with the X-ray source of 50 keV of energy and 200 μA of current with no filter. After acquisition, the reconstructed grey-scale images were converted into binary images using a dynamic threshold of 33–255. The binary images were used for morphometric analysis (CT Analyzer v1.12.0.0, SkyScan, Kontich, Belgium) by the quantification of porosity, mean pore size, and mean wall thickness. The image processing and reconstruction software Data Viewer (v1.6.6.0) and CT-Vox (v2.0.0) (SkyScan, Kontich, Belgium) were used to create, visualize, and register 3D virtual models and cross-sectional and longitudinal cuts.

### 2.11. Cytotoxicity Screening

The L929 cell line from mouse was used to assess the cytotoxicity of the different kefiran extracts as described in the ISO 10993-5 (2009). Briefly, 10,000 cells were seeded in each well of a 96-well plate and 4% (*w/v*) kefiran solutions were diluted in 0.9% (*w/v*) of NaCl, as specified in ISO 10993-12 (2012). After 24 h, the cell culture medium was substituted for each kefiran solution diluted in culture medium at a final concentration of 1% (*v/v*). The culture medium was composed of low-glucose Dulbecco’s Modified Eagle Medium, DMEM (Sigma Aldrich, St. Louis, MO, USA) supplemented with 10% (*v/v*) Fetal Bovine Serum, FBS (Invitrogen, Carlsbad, CA, USA) and 1% (*v/v*) antibiotic/antimycotic (Invitrogen). Two samples composed of only culture medium and the prepared NaCl diluted in culture medium were used as negative controls. Triton X-100 (Sigma-Aldrich, St. Louis, MO, USA) at a concentration of 1% (*v/v*) in culture medium was used as a positive control. Cultures were kept under a humidified atmosphere of 5% (*v/v*) CO_2_ in air at 37 °C. The same conditions were used to assess the cytotoxicity of the different kefiran scaffolds by seeding a density of 1 × 10^5^ cells/cm^2^ on top of each scaffold.

At 24 h, 48 h, and 72 h of culture, cell growth and proliferation were assessed through the AlamarBlue^®^ cell viability assay (Bio-Rad, Amadora, Portugal) and total double-stranded DNA (dsDNA) quantification. At each time point, cells were incubated for 4 h at 37 °C with 10% (*v/v*) of AlamarBlue^®^ reagent. Afterwards, the supernatants were collected, and fluorescence was measured with Ex/Em at 530/590 nm. Each sample and experimental control were performed in triplicate, and culture medium with AlamarBlue^®^ reagent was used as negative control.

For dsDNA quantification, cells were incubated for 1 h at 37 °C in ultrapure water and then stored at −80 °C until analyzed. The Quant-iT PicoGreen dsDNA kit (Invitrogen, Carlsbad, CA, USA) was used following the manufacturer’s instructions. Briefly, samples were diluted in TE buffer into a 96-well white plate and incubated protected from light for 10 min at room temperature after the addition of Quant-iT PicoGreen dsDNA reagent. Fluorescence was measured using a Biotek Synergy HT microplate reader, quantified with Ex/Em at 480/530 nm and R.F.U. were converted into ng/mL using a standard DNA curve in the range of 1–2000 ng/mL. AlamarBlue^®^ and dsDNA quantification data are shown as mean ± standard deviation (*n* = 3).

### 2.12. Statistical Analysis

In the in vitro studies, a two-way ANOVA followed by Tukey’s multiple comparisons test was performed using GraphPad Prism version 8.0.1 for Windows (GraphPad Software, San Diego, CA, USA) to identify statistically significant differences between sample groups.

## 3. Results and Discussion

Three kefiran extracts were obtained through different extraction methods: E1, following the procedure previously described by Radhouani, Goncalves, Maia, Oliveira, and Reis [9]; E2, obtained as E1 but with the inclusion of TCA precipitation of contaminating material (proteins, polypeptides) before kefiran precipitation with ethanol; and E3, extracted as detailed by Pop, Salanţă, Rotar, Semeniuc, Socaciu, and Sindic [24], without either ethanol or TCA precipitation, and with prior freezing of kefir grains. The extraction method with higher yield was E2; from a total of 20 g of kefir grains, E1 and E3 yielded approximate amounts of kefiran (0.730 g and 0.757 g of kefiran, respectively) whilst the final amount of kefiran weighed for E2 was 3.434 g. However, the E2 extract showed to be highly hygroscopic, a characteristic that was not observed for the E1 and E3 extracts. Previous reports of kefiran precipitation with TCA highlight that a great proportion of the EPS (which can represent about 50%) co-precipitates with TCA, resulting in lower kefiran yields observed in techniques containing a step of TCA precipitation [31]. Nonetheless, the inclusion of a TCA precipitation step is reported to be advantageous when aiming for polysaccharide characterization as it allows the recovery of a purer polysaccharide due to the precipitation of contaminating materials such as proteins or polypeptides [31].

The parameters of the extraction method such as the type of the extraction solvent, the size of the raw materials, the extraction temperature and duration, among others, affect the extraction efficiency. It is important to highlight that the extraction methods that were used in this research are green extraction techniques since the kefiran polysaccharides were isolated with hot water. Recently, these green techniques have gained increasing interest principally due to their cost-effectiveness, high extraction yield, and environmentally friendly nature [32].

To explore differences regarding tissue engineering and regenerative medicine applications, the kefiran polysaccharides obtained with each extraction were fully characterized for their structural, physicochemical, and biological properties.

### 3.1. ^1^H-NMR

**Figure 1:**
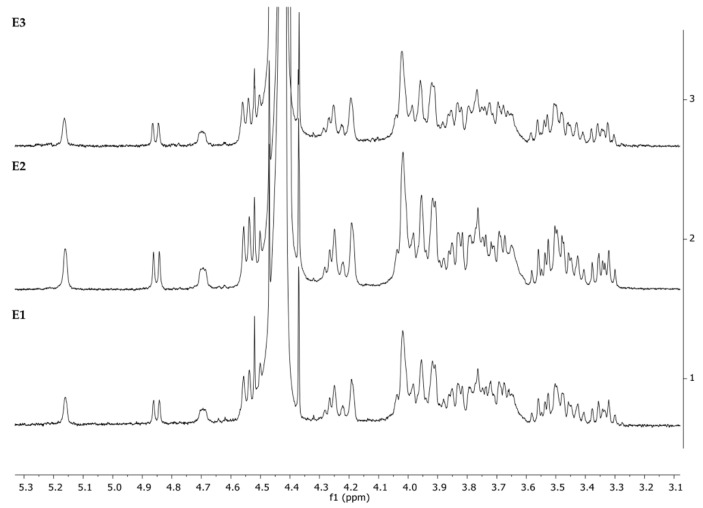
^1^H nuclear magnetic resonance (^1^H-NMR) spectrum of kefiran extracts E1, E2, and E3 in deuterium oxide (D_2_O) at 60 °C.

Nuclear magnetic resonance spectroscopy has been extensively applied as an analytical chemistry technique for the determination of the molecular structure and conformation of polysaccharides [33]. The ^1^H-NMR spectrum of each sample was obtained in D_2_O at 60 °C (Figure 1).

The same peak at around 5.16 ppm was identified in all the extracts (E1, E2, and E3) for an anomeric α hydrogen. The signal occurring in the spectrum should be doublet at 5.16 ppm but appears as a singlet. This could be due to the high temperature (60 °C) used for the acquisition of NMR spectra. Moreover, five signals at the chemical shifts of 4.85 (doublet), 4.69, 4.64, 4.52, and 4.47 for several anomeric β hydrogens were assigned to glucose and galactose rings linked by a β (1–4) glycosidic bond [34]. As expected, we can identify, through NMR spectra, these two saccharides (glucose and galactose), confirming by the way the molecular structure of the kefiran molecule in all extracts.

### 3.2. FTIR

**Figure 2:**
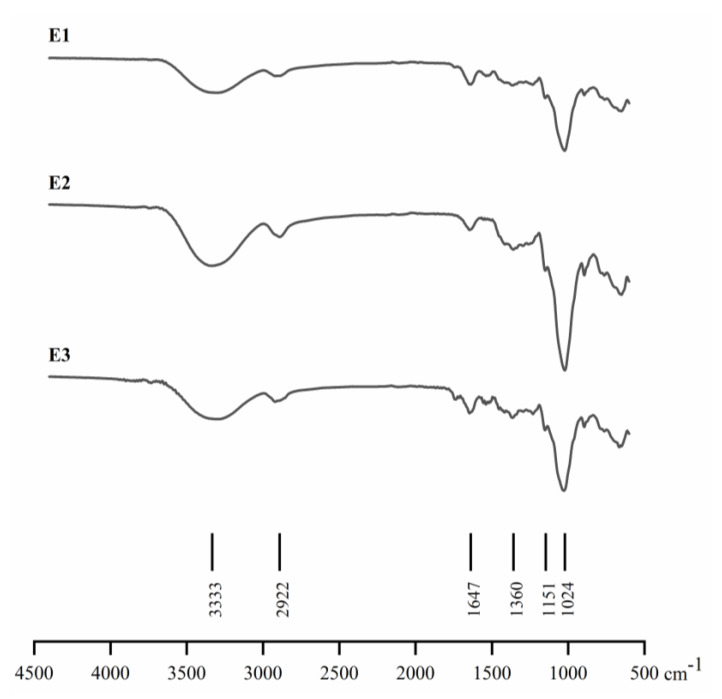
Fourier transform infrared spectroscopy (FTIR) spectra of kefiran extracts E1, E2, and E3.

FTIR analysis is useful to investigate structural changes in biopolymers and has been used to identify not only the fundamental groups present in the kefiran structure but also the reactive functional groups which make kefiran more flexible to modification [8,9,35]. The FTIR spectrum of the different kefiran extracts (Figure 2) showed to be similar and in accordance with those previously reported for kefiran by Radhouani, Goncalves, Maia, Oliveira, and Reis [9], Pop, Salanţă, Rotar, Semeniuc, Socaciu, and Sindic [24] and others. Four major absorption zones were identified: the large absorption band region detected at around 3333 cm^−1^ is attributed to hydroxyl groups (O–H), as a result of the inter- and intra-molecular interactions of the polysaccharide chains. The band identified at around 2922 cm^−1^ corresponds to a C–H stretching vibration zone, and the peak intensity reduction is related to disruption of the kefiran structure due to the water molecules whose presence masked the C–H bond of the carbohydrate rings, thus reducing the contribution of C–H absorbance bands. The bands in the region 1700–1300 cm^−1^ are attributed to the bending mode of O–H; this provides relevant information for industrial applications, since the relative absorption intensities depend on kefiran extraction quality. It is important to highlight that the extracts E1 and E3 showed a band near 1600 cm^−1^, which can be attributed to the N–H bending vibration in primary amine perhaps due to the presence of residual protein. On the contrary, this band was not present in the extract E2, which could be explained by the fact that the extraction process used trichloroacetic acid (TCA). It is well known that this reagent is a very effective protein-precipitating agent and is considered one of the most efficient for precipitating proteins.

The last region, 1200–800 cm^−1^, dominated by ring vibrations overlapped with stretching vibrations of C–OH side groups and C–O–C glycosidic band vibrations, indicates the presence of glucose and galactose of the pure kefiran structure. The structural information and the signal assignments obtained through the ^1^H-NMR and FTIR spectra were furthermore confirmed with the literature spectra of the kefiran polysaccharide [36].

### 3.3. GPC-SEC

Gel permeation/size exclusion chromatography (GPC/SEC) is routinely used to study the molecular weight and structural characteristics of polymers and is useful for polysaccharide characterization. In this study, the Mw of the different kefiran extracts (E1, E2, and E3) was determined by GPC-SEC (Table 1). Results showed that each kefiran extraction method impacted differently the obtained polysaccharide, with E1 yielding the polymer with highest Mw, around 3000 kDa. The other obtained polymers showed significantly lower Mw with values around 1250 kDa for E3 and less than 400 kDa for E2. The kefiran polysaccharide recovered before by Radhouani, Goncalves, Maia, Oliveira, and Reis [9] following the same procedure that was used for the isolation of E1 in this study revealed through SEC a Mn of 357 kDa and Mw of 534 kDa, with a PDI of 1.49; here, a higher molecular weight polymer was recovered, showing also a more uniform chain length as the PDI value is closer to 1. It is important to highlight that those polysaccharides with high molecular weights are normally characterized by a low solubility since disentanglement from the particle surface and consequent dispersion to the main solution will take longer for a large molecule when compared to a smaller one [37]. Moreover, it has been described those polymers with high polydispersity dissolve faster than monodisperse ones [37], a situation also observed in our study.

In the previous work of Pop, Salanţă, Rotar, Semeniuc, Socaciu, and Sindic [24], the Mw of the obtained polysaccharides was between 2.4 × 10^3^ kDa and 1.5 × 10^4^ kDa, being the values dependant on differences in extraction conditions; following the same conditions that were used for E3 isolation (i.e., 80 °C, 30 min), Pop, Salanţă, Rotar, Semeniuc, Socaciu, and Sindic [24] recovered a kefiran polysaccharide with much higher Mw (15,204.5 kDa).

The molecular weight of a polymer impacts its physical (e.g., viscosity, transition temperature) and mechanical (e.g., strength, toughness, stiffness) properties in a way that the lower the Mw, the lower the transition temperature, viscosity, and mechanical properties of a polymer [38]. Hence, when a material has a low Mw, it flows easier, but presents a weak molecular structure as polymer chains are lightly bonded by weak van der Waals forces and can easily move, leading to low strength. On the other hand, as molecular weight increases, the polymer chains become large and entangled, resulting in increased strength, toughness, and stress crack resistance but also in increased viscosity, making the processing of the polymer more difficult [38]. The physicochemical and biological properties can depend on both Mw and molecular weight distributions. In the case of viscosupplementation therapy, the properties of the gold standard hyaluronic acid have been shown to depend on its Mw and concentration, greatly affecting the viscoelastic properties of the synovial fluids [39]. Biologic evidence regarding the use of either low, moderate or high Mw hyaluronic acid is conflicting; still, the optimum hyaluronic acid Mw to achieve high binding affinity and to stimulate endogenous hyaluronic acid production has been shown to be in the range of 500 to 4000 kDa based on in vitro studies, which supports the use of low and moderate Mw hyaluronic acid formulations [40].

### 3.4. Thermal Properties of Kefiran Extracts

**Figure 3:**
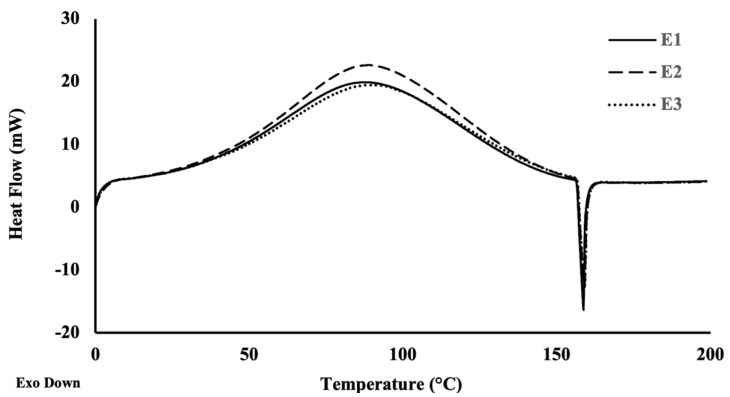
Thermal profile of each kefiran extract measured by DSC. E1, E2, and E3 are represented by the solid, dashed, and dotted lines, respectively.

Thermal analysis is imperative when investigating a broad range of properties of a material. DSC is a simple and precise method for studying the decomposition pattern and the thermal stability of polymers, providing insight into the physical and chemical changes that occur in a polysaccharide during thermal processing [41]. Figure 3 shows the DSC curve of the different kefiran extracts of this study. The outcome of the thermal analysis was similar for all extracts, revealing a small endothermic peak close to 0 °C. This statement is often associated with a melting phase transition that occurs in temperatures below 0 °C, which are endorsed by a small fraction of absorbed or free H_2_O existent on kefiran extracts. Moreover, an endothermic heat flow was observed with a sharp peak at 88.11 °C for E1, at 88.94 °C for E2, and at 90.25 °C for E3, which corresponds to the kefiran polysaccharide melting point.

The calculated enthalpy transitions showed that the kefiran melting process required more energy for E2 than for E1 and E3 (Table 2). At around 156 °C, all samples revealed a sharp exothermic peak corresponding to the degradation of the kefiran samples (Figure 3).

Previous studies using DSC analysis reported higher kefiran melting temperatures, around 99 °C for kefiran extracts [4] and a few degrees above 100 °C for kefiran dense films and porous scaffolds, with melting enthalpies of 355.2 and 294.8 and J/g, respectively [42]. Wang, et al. [43] reported a melting temperature around 93 °C with an endothermic enthalpy change of 249.7 J/g for kefiran produced by *L. kefiranofaciens* ZW3 from Tibet kefir. A melting point of 131.46 °C was reported by Chen et al. [44] for kefiran obtained from Tibetan kefir grains being the enthalpy change 209.6 J/g. In contrary to the extracts E1 and E3 that presented enthalpy changes of 426 J/g, E2 extract showed the highest enthalpy change (523.7 J/g). This statement, in accordance with the literature, could be explained by its lower molecular weight (E2, 400 kDa) and resulting in a lower chain rigidity [45]. Furthermore, when we compare the different spectra, it is possible to point out that the extracts E1, E2, and E3 presented similar profiles; however, through FTIR spectra, the extraction 2 seems to provide kefiran polysaccharide with a higher purity.

Nonetheless, the DSC system used and the sample’s mass, form, density, crucible contact, gas atmosphere, and heating rate are some examples of variables that are known to impact the time and temperature at which the phase transition reaches its maximum. Moreover, as a powerful analytical tool, DSC is able of clarifying the factors that are involved in the folding and stability of pharmaceutical molecules [46].

### 3.5. Kefiran Injectability

Injectable therapeutics assisted by engineered biomaterials have been receiving increasing interest due to the tendency to make clinical practice regenerative and minimally invasive; by offering the possibility to reach deeper, harder to access anatomical locations, and to repair lesions with irregular shapes, these biomaterials reveal immense translational potential [47]. Injectability denotes how a formulation performs during injection, representing a key performance indicator for parenteral dosage forms [48]. The injectability assessment of the different kefiran extracts showed that in 1% (*w/v*) solutions, E1 and E3 required higher extrusion force (13.5 ± 4.7 N and 15.3 ± 5.5 N, respectively) than E2 (6.8 ± 0.3 N), which was near to the measured injectability of water (6.5 ± 0.2 N). This could be explained by the fact that the natural polymer from E1 had a more elastic (or gel-like) behavior compared to the viscous (liquid-like) behavior at a low frequency of E2. Besides the similarity of the flow curves obtained between E2 and E3, an important factor is that the E1 and E3 presented the highest Mw, 3000 kDa and 1250 kDa, respectively, while E2 only showed a Mw of 400 kDa. Thus, the Mw highly affected the needed injectability force but not the shear flow profile. It is well known that a polymer’s molecular weight impacts its physical and mechanical properties. A previously reported kefiran extract obtained through the same method as E1 and using the same injectability measurement setting required only 1 N of force for injection [9]. However, the obtained values are close to that measured in the same conditions for hyaluronic acid (11.3 N) [9], which is considered the gold standard viscosupplement, presently used as a commercial injectable biomaterial and a major player in the category of new injectable hydrogels for knee osteoarthritis treatment [47,49].

### 3.6. Rheological Properties of Kefiran Extracts and Scaffolds

**Figure 4:**
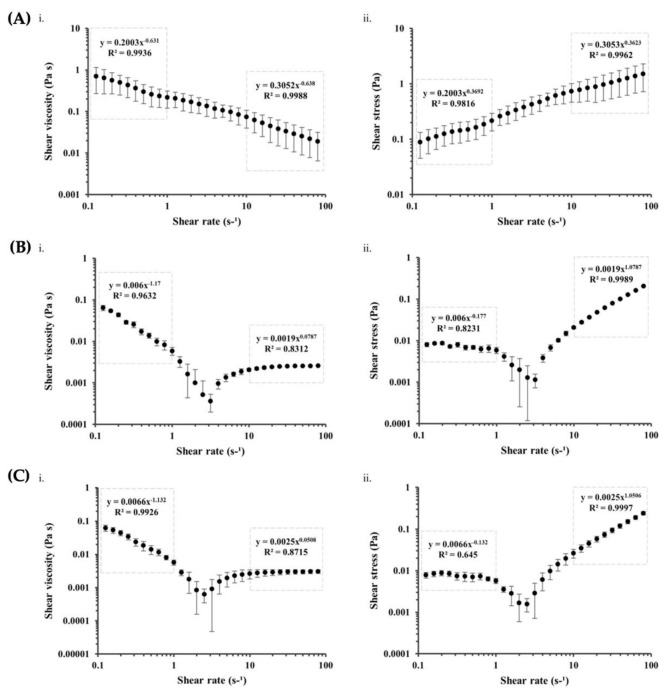
Rheological flow curves of shear viscosity (**i**) and shear stress (**ii**) versus shear rate of kefiran extracts E1 (**A**), E2 (**B**), and E3 (**C**) and respective fitted power-law equations from different power-law regions identified from 0.1 to 1 s^−1^ and 10 to 100 s^−1^.

To simulate physiological conditions, the mechanical tests for each kefiran extract and scaffold were performed at 37 °C. The flow behavior of each kefiran extract is represented in Figure 4, showing shear viscosity (η) and shear stress (σ) values measured as a function of the shear rate (γ). For tissue engineering applications of injectable biomaterials, it is essential to understand how their viscosity depends on the shear rate since it is highly desirable to have low resistance during injection, meaning that the viscosity of these materials should rapidly decrease when subjected to increasing the shear rate [50]. All the tested kefiran extracts show this behavior. For E1 (Figure 4A), a power law region can be observed through the full range of the measured shear rate; this typically describes shear thinning behavior, also known as pseudoplastic flow, which is the most common type of non-Newtonian behavior where fluid viscosity decreases, and shear stress rises with increasing shear rate. Most polymer solutions exhibit shear thinning behavior and these results are in accordance with those previously described for kefiran extracted through the same procedure and analyzed in 1% and 10% (*w/v*) solutions at 37 °C [9,15], and others [18,19,20,21,22,23,51,52]. However, for E2 and E3 (Figure 4B,C), this shear thinning behavior is only observed for lower shear rates, showing a shift to an apparent Newtonian behavior that is not observed for E1 in the same shear rate range. High molecular weight polymers such as kefiran are usually intertwined and randomly oriented but start to unwind and align in the direction of flow when sheared, causing viscosity to decrease. Since this is dependant of shear rate, at sufficiently high shear rates, the polymeric chains in the solution will be fully separated and aligned, resulting in a Newtonian state where viscosity becomes independent of shear rate, which reflects the behavior observed for E2 and E3 at shear rates above 10 s^−1^. The power-law Ostwald de Waele model (σ = Kγ^n^ or η = Kγ^n−1^), the most frequently used model for non-Newtonian fluid mechanics, was applied to determine the flow consistency index (K) and flow-behavior index (n), a unitless number indicating the closeness to Newtonian flow (*n* = 1 for Newtonian, *n* < 1 for pseudoplastic/shear thinning, and *n* > 1 for dilatant/shear thickening fluids). The power-law equations of each kefiran extract reveal, for the shear rate range of 0.1 to 1 s^−1^, consistency flow indexes of 0.2 for E1 and of 0.006 for E2 and E3, being all flow behavior indexes far below 1 (0.369, −0.177, and −0.132 for E1, E2, and E3, respectively). For the shear rate range of 10 to 100 s^−1^, consistency flow indexes were of 0.3 for E1 and of 0.0019 for E2 and E3, being the flow behavior indexes 0.362 for E1 but 1.0787 and 1.0506 for E2 and E3, respectively, showing the distinct nearly Newtonian behavior of these samples at higher shear rates. Similar consistency and flow behavior indexes were previously described for kefiran [9,19,20,23,24,52,53]. The viscoelastic properties of all the kefiran extracts, but particularly E1, show their potential for TERM applications, namely as an economical alternative therapy to the traditional hyaluronic acid in restoring the viscoelastic properties of the joint synovial fluid in osteoarthritis cases [9,16,54].

**Figure 5:**
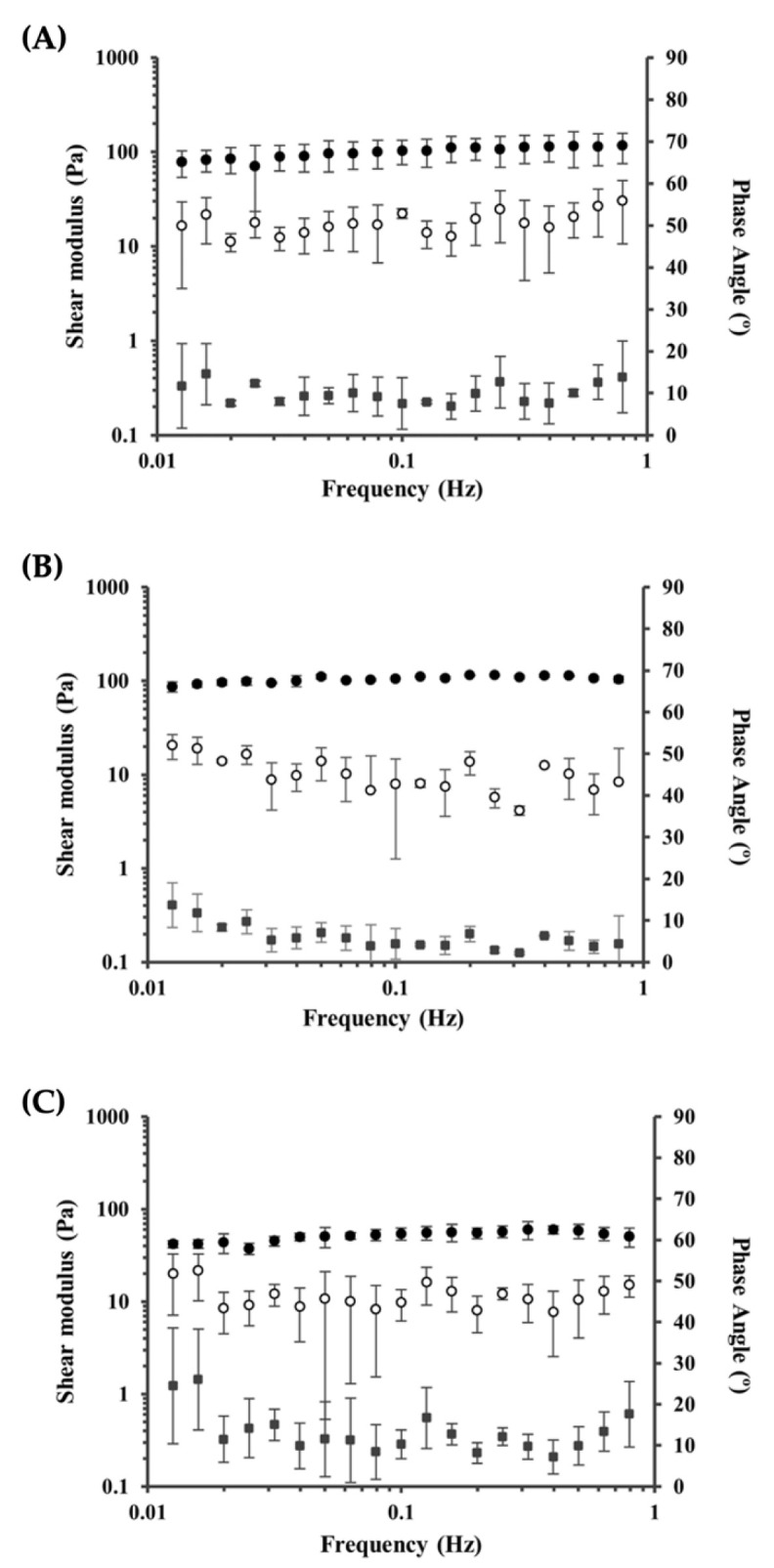
Frequency sweep curves for kefiran scaffolds S1 (**A**), S2 (**B**), and S3 (**C**). The storage and loss moduli are represented by G′ (●) and G″ (○), respectively. The phase-angle (■) is represented on the secondary axis.

Suitable mechanical properties of scaffolds are of major importance for TERM applications, not only to possess sufficient mechanical strength, comparable to that of native tissue to be repaired, but also to deliver a proper microenvironment for cellular growth [55,56]. Indeed, scaffold stiffness and the stresses generated by the occurring cell-scaffold strains have been shown to have a significant impact on cells, particularly in stem cell differentiation [56]. Hence, the viscoelastic behavior is a key parameter to be addressed, particularly at low strain rates and within physiological frequency ranges [56]. Oscillatory experiments were performed on the kefiran scaffolds to study their viscoelasticity though frequency sweep curves starting at 0.01 Hz to 1 Hz and results can be observed in Figure 5.

For all samples analyzed, the storage modulus (G′), which measures the elastic component of the sample, was, as expected, higher than the loss modulus (G′′), which reflects the viscous component. This elastic character of kefiran scaffolds has been previously reported by [16], quantified by a phase angle of 16° ± 0.7°. The phase-angle is a direct measure of viscoelasticity and indicates the phase shift between the input signal (i.e., the applied oscillation) and the output signal, which represents the sample response. Viscoelastic materials fall in the range of 90° (solid) and 0° (liquid), being elastic solids those with values closer to 0° and higher storage modulus (G′) and viscous liquids, those closer to 90° with higher loss modulus (G′′) [57]. In this study, all scaffolds exhibited a similar trend throughout the measured frequency, with phase angle values of 9.06° ± 2.08° for S1, 5.43° ± 1.41° for S2, and 11.23° ± 2.49° for S3. The viscoelastic properties (elastic/gel character) and mechanical stability observed in all kefiran scaffolds are of major importance regarding TERM applicability, proving their potential in this context.

### 3.7. Kefiran Scaffold Microstructure

**Figure 6:**
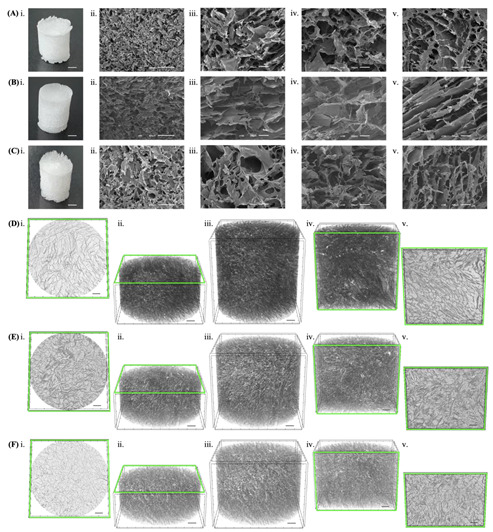
Morphology of kefiran scaffolds obtained from each extract, S1 (**A**,**D**), S2 (**B**,**E**), and S3 (**C**,**F**). (**A**–**C**) show the macroscopic image (**i**) of each scaffold (scale bar: 1 mm) and the SEM microstructures for longitudinal sections at magnifications of ×50 (**ii**) and ×150 (**iii**) and for cross-sectional (**iv**) and uncut (**iii**) views at ×150 of magnification. (**D**–**F**) show 3D reconstructed images of each scaffold using micro-CT: cross-sectional (**i**,**ii**), longitudinal (**iv**,**v**), and fully reconstructed (**iii**) views (scale bars: 500 µm).

**Figure 7:**
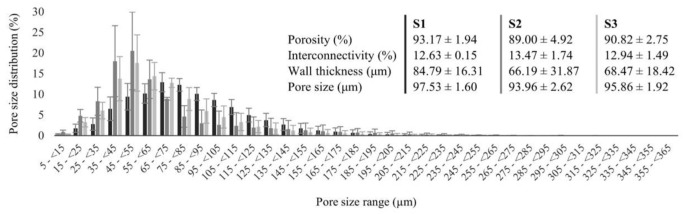
Micro-CT data regarding pore size distribution and structure characterization of the scaffolds obtained from each extract, S1 (black), S2 (dark grey), and S3 (light grey).

The detailed morphology of each kefiran scaffold was analyzed by SEM and micro-CT. A highly porous structure was observed across all samples in the longitudinal sections, cross-sections, and uncut views analyzed by SEM (Figure 6A–C). The interconnectivity and porous structure of all scaffolds was confirmed using micro-CT through 3D reconstructed images of each scaffold (Figure 6D–F). The qualitative and quantitative analysis of the architecture of each scaffold investigated by micro-CT showed that all kefiran scaffolds presented high porosity and a widespread pore size distribution, revealing similar average values that were close to 90% and above 90 µm, respectively (Figure 7). Porosity is considered the key characteristic of TERM scaffolds but since it is determined by open and closed pores of variable size, shape, spatial distribution, and mutual interconnection, accomplishing an accurate comparison of pore size remains a challenge in scaffold materials [58]. When compared to SEM, the gold-standard method for pore size evaluation, micro-CT stands out as an emerging effective method with interesting advantages but can over/underestimate pore size in irregularly shaped pores [58]. Still, the optimal pore size for TERM is tissue specific and consensus regarding the optimal scaffold pore size is yet to be accomplished. Pore sizes ranging from 50 to 710 μm have been suggested for bone regeneration with values around 100 to 350 μm suggested as ideal; also, sizes in the range of 200 to 300 μm are recommended for fibrocartilaginous tissue growth, the optimal pore size for neovascularization is considered 5 μm, while for fibroblast ingrowth, the ideal range of 5 to 15 μm is reported [59,60]. The micro-CT morphometric analysis also revealed that the different scaffolds analyzed in this study presented interesting values regarding wall thickness and interconnectivity (Figure 7).

For all TERM applications, porosity, pore size, wall thickness, and pore interconnectivity are critical scaffold structural properties that directly impact both in vitro and in vivo scaffold functionality as they determine permeability, diffusivity, degradation rate, and elastic modulus of the scaffold, and therefore the biological processes required for tissue regeneration [61,62]. An interconnected porous network enhances cell proliferation and migration through the scaffold and allows nutrient transport and waste removal; hence, the distinct pore analysis parameters have direct influence over cell behavior and define the final mechanical properties of the scaffold [61].

### 3.8. Cytocompatibility of Different Kefiran Extracts and Scaffolds

**Figure 8:**
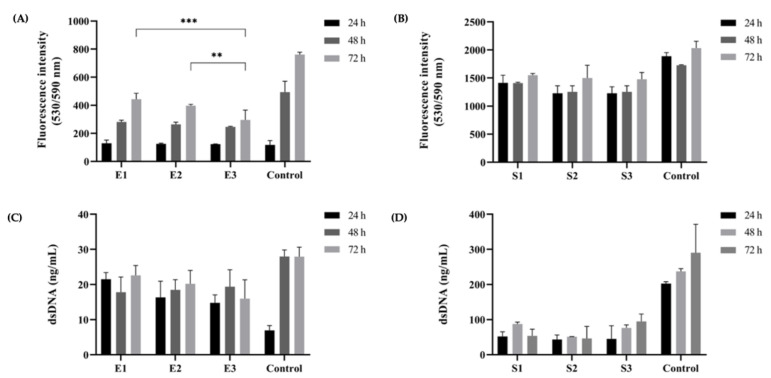
Cytocompatibility of different kefiran extracts and scaffolds. AlamarBlue^®^ quantification indicated statistically significant differences in the metabolic activity of L929 cells exposed to kefiran extracts after 72 h (**A**), but no statistically significant changes were observed between scaffolds (**B**). dsDNA quantification of exposed L929 cells did not show significant changes between extracts (**C**) or scaffolds (**D**) at any timepoint. ** *p* < 0.01; *** *p* < 0.001.

Regarding the viability and proliferation of L929 cells exposed to the different extracts, AlamarBlue^®^ reads only showed significant differences between E1 and E3 (*p* = 0.0002) and E2 and E3 (*p* = 0.0093) after 72 h (Figure 8A). All extracts showed significant differences when compared to the control after 48 h and 72 h (*p* < 0.0001) and significant changes between timepoints were observed for all extracts. Measurements of dsDNA also showed significant differences between all extracts and the control after 24 h and 48 h, and only E3 was significantly different after 72 h. However, no significant changes were observed among E1, E2, and E3 at any timepoint (Figure 8C). Concerning the different scaffolds, no significant changes were observed in the AlamarBlue^®^ reads between S1, S2, and S3 for any timepoint (Figure 8B); varying levels of significance were obtained between each scaffold and the control. Equivalent results were noted for dsDNA measurements (Figure 8D).

The results show that, as expected, none of the kefiran extracts or scaffolds show any cytotoxic effect over L929 cells, and no significant changes were observed that could clearly distinguish them regarding cytocompatibility. It is important to point out that cytocompatibility is the most usually used term to describe suitable biological requirements of a biomaterial used in a medical device. Thus, all the different kefiran extracts and scaffolds, which were cytocompatible and enhanced the cell proliferation, could represent a potential candidate for tissue engineering application. Nonetheless, for TERM applications, biomaterials should not just be biocompatible for cells, but they should act as the extracellular matrix of native tissues, preserving biochemical cues and properties, so these polysaccharide-based materials should improve cellular adhesion, proliferation, and differentiation and at the same time avoid immunological reactions [63]. Thus, it will be interesting for further research, to evaluate the in vitro model of different cell lines incorporated in these three kefiran-based scaffolds.

## 4. Conclusions

High-quality kefiran polysaccharides with suitable yield were obtained through different extraction protocols, and kefiran cryogels were successfully produced from different extracts by freeze-drying. Both products, kefiran extracts and 3D scaffolds, were fully characterized for their structural and physicochemical properties. In general, the three extracts showed thermal stability and desirable viscoelastic properties. One of the three extracts (E1) stood out and demonstrated to have the greatest physical and mechanical properties, similar to those of hyaluronic acid, the gold standard viscosupplement presently used as a commercial injectable biomaterial and a major player in the category of new injectable hydrogels for osteoarthritis treatment. All kefiran extracts and scaffolds proved to be non-cytotoxic over L929 cells. Furthermore, the developed scaffolds showed mechanical stability, elastic behavior, and high porosity which are characteristics of major importance regarding TERM applicability.

The current research demonstrated that the application of these developed products is conditioned by the employed extraction method. While the three kefiran products showed interesting structural, physicochemical, and biological properties, each one of them could be used for a certain biomedical application.

Nowadays, it is of great significance to develop effective and selective methods for the extraction of bioactive polymers to develop functional biomaterials. For future research, since the extraction technique was optimized, it will be a great interest and huge challenge to use more advanced technologies, such as cell-electrospinning, 3D printing, microfluidics, among others, to develop smart biomaterials that will better mimic the properties of the native tissues.

## Figures and Tables

**Table 1:** Gel permeation/size exclusion chromatography (GPC/SEC) data obtained for kefiran extracts E1, E2, and E3: number-average molecular weight (Mn), molecular weight of the peak maxima (Mp), weight-average molecular weight (Mw), polydispersity index (PDI), and intrinsic viscosity (IV).

	Mn (kDa)	Mp (kDa)	Mw (kDa)	PDI (Mw/Mn)	IV (dL/g)
E1	2830.00 ± 647.18	3038.67 ± 490.27	2994.33 ± 674.84	1.058 ± 0.008	1
E2	90.77 ± 6.69	361.87 ± 13.77	335.17 ± 9.05	3.70 ± 0.21	1
E3	1035.00 ± 76.22	1241.33 ± 58.94	1244.67 ± 74.45	1.204 ± 0.054	1

**Table 2:** Melting temperatures and associated enthalpy changes in DSC thermograms obtained for the different kefiran extracts.

	Peak (°C)	Onset (°C)	Δ*H* (J/g)	Heat Flow (mW)
E1	88.11	32.74	426.6	19.88
E2	88.94	35.11	523.7	22.61
E3	90.25	33.39	426.2	19.47

## Data Availability

All data generated/analyzed throughout this research are included in this article.

## References

[1] Sood A., Gupta A., Agrawal G. (2021). Recent advances in polysaccharides based biomaterials for drug delivery and tissue engineering applications. Carbohydr. Polym. Technol. Appl..

[2] Litowczenko J., Woźniak-Budych M.J., Staszak K., Wieszczycka K., Jurga S., Tylkowski B. (2021). Milestones and current achievements in development of multifunctional bioscaffolds for medical application. Bioact. Mater..

[3] Barclay T.G., Day C.M., Petrovsky N., Garg S. (2019). Review of polysaccharide particle-based functional drug delivery. Carbohydr. Polym..

[4] Souza P.R., de Oliveira A.C., Vilsinski B.H., Kipper M.J., Martins A.F. (2021). Polysaccharide-Based Materials Created by Physical Processes: From Preparation to Biomedical Applications. Pharmaceutics.

[5] Bačáková L., Novotná K., Pařízek M. (2014). Polysaccharides as cell carriers for tissue engineering: The use of cellulose in vascular wall reconstruction. Physiol. Res..

[6] Arslan S. (2015). A review: Chemical, microbiological and nutritional characteristics of kefir. CyTA J. Food.

[7] Cottet C., Ramirez-Tapias Y.A., Delgado J.F., de la Osa O., Salvay A.G., Peltzer M.A. (2020). Biobased Materials from Microbial Biomass and Its Derivatives. Materials.

[8] Moradi Z., Kalanpour N. (2019). Kefiran, a branched polysaccharide: Preparation, properties and applications: A review. Carbohydr. Polym..

[9] Radhouani H., Goncalves C., Maia F.R., Oliveira J.M., Reis R.L. (2018). Kefiran biopolymer: Evaluation of its physicochemical and biological properties. J. Bioact. Compat. Polym..

[10] Azizi N.F., Kumar M.R., Yeap S.K., Abdullah J.O., Khalid M., Omar A.R., Osman M.A., Mortadza S.A.S., Alitheen N.B. (2021). Kefir and Its Biological Activities. Foods.

[11] Lopresti F., Campora S., Tirri G., Capuana E., Carfì Pavia F., Brucato V., Ghersi G., La Carrubba V. (2021). Core-shell PLA/Kef hybrid scaffolds for skin tissue engineering applications prepared by direct kefiran coating on PLA electrospun fibers optimized via air-plasma treatment. Mater. Sci. Eng. C.

[12] Salari A., Hashemi M., Afshari A. (2022). Functional Properties of Kefiran in the Medical Field and Food Industry. Curr. Pharm. Biotechnol..

[13] Correia S., Oliveira J.M., Radhouani H., Reis R.L., Oliveira J., Radhouani H., Reis R.L. (2020). Kefiran in Tissue Engineering and Regenerative Medicine. Polysaccharides of Microbial Origin: Biomedical Applications.

[14] Yap J.X., Leo C.P., Mohd Yasin N.H., Show P.L., Chu D.T., Singh V., Derek C.J.C. (2022). Recent advances of natural biopolymeric culture scaffold: Synthesis and modification. Bioengineered.

[15] Radhouani H., Goncalves C., Reis R.L., Oliveira J.M. (2017). Kefiran for Use in Regenerative Medicine and/or Tissue Engineering.

[16] Radhouani H., Bicho D., Goncalves C., Maia F.R., Reis R.L., Oliveira J.M. (2019). Kefiran cryogels as potential scaffolds for drug delivery and tissue engineering applications. Mater. Today Commun..

[17] Radhouani H., Correia S., Goncalves C., Reis R.L., Oliveira J.M. (2021). Synthesis and Characterization of Biocompatible Methacrylated Kefiran Hydrogels: Towards Tissue Engineering Applications. Polymers.

[18] Esnaashari S.S., Rezaei S., Mirzaei E., Afshari H., Rezayat S.M., Faridi-Majidi R. (2014). Preparation and characterization of kefiran electrospun nanofibers. Int. J. Biol. Macromol..

[19] Piermaria J.A., de la Canal M.L., Abraham A.G. (2008). Gelling properties of kefiran, a food-grade polysaccharide obtained from kefir grain. Food Hydrocoll..

[20] Zavala L., Roberti P., Piermaria J.A., Abraham A.G. (2015). Gelling ability of kefiran in the presence of sucrose and fructose and physicochemical characterization of the resulting cryogels. J. Food Sci. Technol..

[21] Sabatino M.A., Carfi Pavia F., Rigogliuso S., Giacomazza D., Ghersi G., La Carrubba V., Dispenza C. (2020). Development of injectable and durable kefiran hydro-alcoholic gels. Int. J. Biol. Macromol..

[22] Ghasemlou M., Khodaiyan F., Jahanbin K., Gharibzahedi S.M., Taheri S. (2012). Structural investigation and response surface optimisation for improvement of kefiran production yield from a low-cost culture medium. Food Chem..

[23] Exarhopoulos S., Raphaelides S.N., Kontominas M.G. (2018). Flow behavior studies of kefiran systems. Food Hydrocoll..

[24] Pop C.R., Salanţă L., Rotar A.M., Semeniuc C.A., Socaciu C., Sindic M. (2016). Influence of extraction conditions on characteristics of microbial polysaccharide kefiran isolated from kefir grains biomass. J. Food Nutr. Res..

[25] Zhang Q.W., Lin L.G., Ye W.C. (2018). Techniques for extraction and isolation of natural products: A comprehensive review. Chin. Med..

[26] Joyce K., Fabra G.T., Bozkurt Y., Pandit A. (2021). Bioactive potential of natural biomaterials: Identification, retention and assessment of biological properties. Signal Transduct. Target. Ther..

[27] Ullah S., Khalil A.A., Shaukat F., Song Y. (2019). Sources, Extraction and Biomedical Properties of Polysaccharides. Foods.

[28] Karvinen J., Ihalainen T.O., Calejo M.T., Jonkkari I., Kellomaki M. (2019). Characterization of the microstructure of hydrazone crosslinked polysaccharide-based hydrogels through rheological and diffusion studies. Mater. Sci. Eng. C Mater. Biol. Appl..

[29] Welzel P.B., Prokoph S., Zieris A., Grimmer M., Zschoche S., Freudenberg U., Werner C. (2011). Modulating Biofunctional starPEG Heparin Hydrogels by Varying Size and Ratio of the Constituents. Polym. Basel.

[30] Suriano R., Griffini G., Chiari M., Levi M., Turri S. (2014). Rheological and mechanical behavior of polyacrylamide hydrogels chemically crosslinked with allyl agarose for two-dimensional gel electrophoresis. J. Mech. Behav. Biomed..

[31] Rimada P., Abraham A. (2003). Comparative study of different methodologies to determine the exopolysaccharide produced by kefir grains in milk and whey. Le Lait.

[32] Carpentieri S., Soltanipour F., Ferrari G., Pataro G., Donsì F. (2021). Emerging Green Techniques for the Extraction of Antioxidants from Agri-Food By-Products as Promising Ingredients for the Food Industry. Antioxidants.

[33] Yao H.Y., Wang J.Q., Yin J.Y., Nie S.P., Xie M.Y. (2021). A review of NMR analysis in polysaccharide structure and conformation: Progress, challenge and perspective. Food Res. Int..

[34] Kosaka A., Aida M., Katsumoto Y. (2015). Reconsidering the activation entropy for anomerization of glucose and mannose in water studied by NMR spectroscopy. J. Mol. Struct..

[35] Jenab A., Roghanian R., Emtiazi G. (2015). Encapsulation of Platelet in Kefiran Polymer and Detection of Bioavailability of Immobilized Platelet in Probiotic Kefiran as a New Drug for Surface Bleeding. J. Med. Bacteriol..

[36] Pop C.R., Apostu S., Rotar A.M., Semeniuc C.A., Sindic M., Mabon N. (2013). FTIR spectroscopic characterization of a new biofilm obtained from kefiran. J. Agroaliment. Processes Technol..

[37] Mark Q.G., Xinzhong H., Changlu W., Lianzhong A. (2017). Polysaccharides: Structure and Solubility. Solubility Polysacch..

[38] Balani K., Verma V., Agarwal A., Narayan R., Balani K., Verma V.A.A., Narayan R. (2014). Physical, Thermal, and Mechanical Properties of Polymers. Biosurfaces.

[39] Li C., Cao Z., Li W., Liu R., Chen Y., Song Y., Liu G., Song Z., Liu Z., Lu C. (2020). A review on the wide range applications of hyaluronic acid as a promising rejuvenating biomacromolecule in the treatments of bone related diseases. Int. J. Biol. Macromol..

[40] Shewale A.R., Barnes C.L., Fischbach L.A., Ounpraseuth S.T., Painter J.T., Martin B.C. (2017). Comparison of Low-, Moderate-, and High-Molecular-Weight Hyaluronic Acid Injections in Delaying Time to Knee Surgery. J. Arthroplast..

[41] Bothara S.B., Singh S. (2012). Thermal studies on natural polysaccharide. Asian Pac. J. Trop. Biomed..

[42] Montesanto S., Calo G., Cruciata M., Settanni L., Brucato V.B., La Carrubba V. (2016). Optimization of Environmental Conditions for Kefiran Production by Kefir Grain as Scaffold for Tissue Engineering. Chem. Eng. Trans..

[43] Wang Y., Ahmed Z., Feng W., Li C., Song S. (2008). Physicochemical properties of exopolysaccharide produced by Lactobacillus kefiranofaciens ZW3 isolated from Tibet kefir. Int. J. Biol. Macromol..

[44] Chen Z.N., Shi J.L., Yang X.J., Nan B., Liu Y., Wang Z.F. (2015). Chemical and physical characteristics and antioxidant activities of the exopolysaccharide produced by Tibetan kefir grains during milk fermentation. Int. Dairy J..

[45] McGonigle E.A., Cowie J., Arrighi V., Pethrick R. (2005). Enthalpy relaxation and free volume changes in aged styrene copolymers containing a hydrogen bonding co-monomer. J. Mater. Sci..

[46] Steinmann W., Walter S., Beckers M., Seide G., Gries T. (2013). Thermal Analysis of Phase Transitions and Crystallization in Polymeric Fibers. Applications of Calorimetry in a Wide Context.

[47] Zhou H., Liang C., Wei Z., Bai Y., Bhaduri S.B., Webster T.J., Bian L., Yang L. (2019). Injectable biomaterials for translational medicine. Mater. Today.

[48] Cilurzo F., Selmin F., Minghetti P., Adami M., Bertoni E., Lauria S., Montanari L. (2011). Injectability evaluation: An open issue. AAPS PharmSciTech.

[49] Fakhari A., Berkland C. (2013). Applications and emerging trends of hyaluronic acid in tissue engineering, as a dermal filler and in osteoarthritis treatment. Acta Biomater..

[50] Borzacchiello A., Sala F.D., Ambrosio L., Tanzi M., Fare S. (2017). 10-Rheometry of polymeric biomaterials. Characterization of Polymeric Biomaterials.

[51] Bagherian Far M., Ziyadi H. (2016). Fabrication of Polyvinyl Alcohol/Kefiran Nanofibers Membrane Using Electrospinning. J. Pharm. Health Sci..

[52] Ghasemlou M., Khodaiyan F., Oromiehie A. (2011). Rheological and structural characterisation of film-forming solutions and biodegradable edible film made from kefiran as affected by various plasticizer types. Int. J. Biol. Macromol..

[53] Piermaria J.A., Pinotti A., Garcia M.A., Abraham A.G. (2009). Films based on kefiran, an exopolysaccharide obtained from kefir grain: Development and characterization. Food Hydrocoll..

[54] Marangoni Junior L., Vieira R.P., Anjos C.A.R. (2020). Kefiran-based films: Fundamental concepts, formulation strategies and properties. Carbohydr. Polym..

[55] Prasadh S., Wong R.C.W. (2018). Unraveling the mechanical strength of biomaterials used as a bone scaffold in oral and maxillofacial defects. Oral Sci. Int..

[56] Kocen R., Gasik M., Gantar A., Novak S. (2017). Viscoelastic behaviour of hydrogel-based composites for tissue engineering under mechanical load. Biomed. Mater..

[57] Gonçalves C., Silva S.S., Gomes J.M., Oliveira I.M., Canadas R.F., Maia F.R., Radhouani H., Reis R.L., Oliveira J.M. (2020). Ionic Liquid-Mediated Processing of SAIB-Chitin Scaffolds. ACS Sustain. Chem. Eng..

[58] Bartos M., Suchy T., Foltan R. (2018). Note on the use of different approaches to determine the pore sizes of tissue engineering scaffolds: What do we measure?. Biomed. Eng. Online.

[59] Kramschuster A., Turng L.-S., Ebnesajjad S. (2013). 17-Fabrication of Tissue Engineering Scaffolds. Handbook of Biopolymers and Biodegradable Plastics.

[60] Gorth D., Webster T.J., Lysaght M., Webster T.J. (2011). 10-Matrices for tissue engineering and regenerative medicine. Biomaterials for Artificial Organs.

[61] Loh Q.L., Choong C. (2013). Three-dimensional scaffolds for tissue engineering applications: Role of porosity and pore size. Tissue Eng. Part B Rev..

[62] Poh P.S.P., Valainis D., Bhattacharya K., van Griensven M., Dondl P. (2019). Optimization of Bone Scaffold Porosity Distributions. Sci. Rep..

[63] Puertas-Bartolomé M., Mora-Boza A., García-Fernández L. (2021). Emerging Biofabrication Techniques: A Review on Natural Polymers for Biomedical Applications. Polymers.

